# Generalizability of Alzheimer’s disease plasma biomarkers phospho‐Tau and beta amyloid in individuals of diverse genetic ancestries

**DOI:** 10.1002/alz.092669

**Published:** 2025-01-09

**Authors:** Anthony J. Griswold, Farid Rajabli, Tianjie Gu, Jamie Arvizu, Charles G. Golightly, Patrice L. Whitehead, Kara L. Hamilton‐Nelson, Larry D. Adams, Katrina Celis, Katrina Celis, Jose Javier Sanchez, Pedro R. Mena, Takiyah D. Starks, Maryenela Z. Illanes‐Manrique, Concepcion Silva‐Vergara, Michael L. Cuccaro, Jeffery M. Vance, Briseida E. Feliciano‐Astacio, Mario Cornejo‐Olivas, Goldie S. Byrd, Gary Beecham, Jonathan L. Haines, Margaret A Pericak‐Vance

**Affiliations:** ^1^ John P. Hussman Institute for Human Genomics, University of Miami Miller School of Medicine, Miami, FL, USA, Miami, FL USA; ^2^ Dr. John T. Macdonald Foundation Department of Human Genetics, University of Miami Miller School of Medicine, Miami, FL USA; ^3^ John P. Hussman Institute for Human Genomics, University of Miami Miller School of Medicine, Miami, FL USA; ^4^ Maya Angelou Center for Health Equity, Wake Forest University School of Medicine, Winston‐Salem, NC USA; ^5^ Neurogenetics Research Center, Instituto Nacional de Ciencias Neurológicas, Lima Peru; ^6^ Universidad Central del Caribe, Bayamón, PR USA; ^7^ Department of Population and Quantitative Health Sciences, Institute for Computational Biology, Case Western Reserve University, Cleveland, OH USA; ^8^ 1501 NW 10th Avenue, Miami, FL USA

## Abstract

**Background:**

Plasma concentrations of phosphorylated threonine‐181 of Tau (pTau181) and the ratio of amyloid beta isoforms Aβ42/Aβ40 are biomarkers for differential diagnosis and preclinical detection of Alzheimer disease (AD). However, assessment of the utility of these biomarkers has been in non‐Hispanic, European individuals. Given differences in AD risk across populations, generalizability of these findings is not assured in individuals of diverse ancestries. Here we evaluate the levels of plasma pTau181 and Aβ42/Aβ40 and assess their utility in discriminating clinically diagnosed AD from cognitively unimpaired (CU), age‐matched controls in ancestrally diverse, admixed cohorts.

**Method:**

We measured pTau181 and Aβ42/Aβ40 with Simoa chemistry using the pTau181 AdvantageV2 and NEUROLOGY 3‐PLEX A assays, respectively. Our cohorts consisted of: 923 African Americans (319 AD, 604 CU), 149 Peruvians (49 AD, 100 CU), and 667 Caribbean Hispanics (613 Puerto Ricans (288 AD, 325 CU) and 54 Cubans (24 AD, 30 CI)). Linear mixed‐effect regression models adjusted for age, sex, population substructure and relatedness followed by Bonferroni correction was applied to identify biomarker differences. Diagnostic performance and receiver operator characteristic (ROC) curves were created from logistic regression models.

**Result:**

Plasma pTau181 concentrations did not differ across any of the cohorts within AD or CU. There was a clear elevation of pTau181 concentration in AD compared to CU (p < 1.7x10^‐13^) taking into account all individuals and in each cohort separately (African Americans, p =1.9x10^‐8^; Caribbean Hispanics, p=3.1x10^‐7^; Peruvians, p=0.05). There was no significant difference in the plasma Aβ42/Aβ40 ratio, however there was a trend towards a decreasing ratio in AD. Using the area under the ROC, pTau181 was more accurate at predicting status than the Aβ42/Aβ40 ratio, but the classification improved when both biomarkers were combined.

**Conclusion:**

These results suggest AD biomarkers are generalizable across ancestries, with baseline values being consistent across diverse populations. Ultimately, combining genomic, biomarker, and social and environmental data from diverse individuals will increase understanding of genetic risk and refine clinical diagnoses in individuals of diverse ancestries.

